# Sacubitril/valsartan in heart failure and end‐stage renal insufficiency

**DOI:** 10.1002/ehf2.12544

**Published:** 2019-10-30

**Authors:** Alex Heyse, Lynn Manhaeghe, Elien Mahieu, Céline Vanfraechem, Frederik Van Durme

**Affiliations:** ^1^ Department, of Cardiology AZ Glorieux Ronse Belgium; ^2^ Department of Nephrology AZ Glorieux Ronse Belgium

**Keywords:** Sacubitril/valsartan, ARNI, Heart failure, Dialysis, Chronic kidney disease

## Abstract

The aim of this report is to describe the feasibility and tolerability of medical treatment with sacubitril/valsartan in a patient treated with hemodialysis. We describe the case of a 67‐year‐old man with heart failure with reduced ejection fraction due to an ischemic cardiomyopathy and renal insufficiency undergoing hemodialysis. Because of worsening heart failure with no other therapeutic options, a treatment with sacubitril/valsartan was started. Although this patient had a very low systolic blood pressure, he could tolerate a moderate dose of 49/51 mg twice daily. After initiation of sacubitril/valsartan, there was a symptomatic improvement with a clear reduction NT‐proBNP, accompanied by a decrease in filling pressures. In conclusion, in this patient with severe heart failure undergoing hemodialysis, treatment with sacubitril/valsartan was feasible, safe, and improved heart failure symptoms.

## Introduction

Compared to angiotensin converting enzyme (ACE) inhibition, the angiotensin‐receptor neprilysin inihibitor (ARNI) sacubitril/valsartan has demonstrated a reduction in both mortality and hospitalization for heart failure in a symptomatic patient population with reduced EF less than 35%.^1^ Beyond these hard end points, sacubitril/valsartan decreased symptoms and improved physical limitations of heart failure. Yet patients with severe renal insufficiency with a glomerular filtration rate (GFR) below 30 ml per minute per 1.73 m^2^ of body‐surface area at screening or randomization were excluded from the PARADIGM‐HF trial.^1^ Patients with severe chronic kidney disease are at very high risk for cardiac events.^2^ Conversely, patients with heart failure frequently have reduced renal function. Data on tolerability and outcome of patients with severe renal insufficiency treated with sacubitril/valsartan are lacking.

## Case report

A 67‐year‐old man treated with hemodialysis was evaluated for worsening heart failure with reduced ejection fraction (EF). Eighteen years ago, he experienced an anterior ST‐elevation myocardial infarction (STEMI) treated by percutaneous coronary intervention (PCI) of the proximal left anterior descending (LAD). He was a former smoker and had both diabetes Type 2 and hypercholesterolemia. Ten years ago, he underwent coronary angiography showing progression of coronary artery disease with occlusion of the LAD and the right coronary artery (RCA). Bypass grafting was performed with left internal mammary artery (LIMA) to the LAD and a saphenous vein graft to the RCA. Five years ago, he had a posterolateral STEMI due to a circumflex (CX) occlusion, treated by PCI of the proximal CX and marginal branch. The STEMI was complicated by an acute pulmonary edema and hypotension. The left ventricular (LV) function was severely depressed with an EF 34% and high filling pressures. At that moment, a mild to moderate renal impairment was present with an estimated glomerular filtration rate (eGFR) of 52 ml/min per 1.73 m^2^. Heart failure treatment was optimized, but due to hypotension, only a moderate dose of bisoprolol (5 mg) and ramipril (5 mg) was tolerated. He received both bumetanide and spironolactone. Despite optimal medical therapy, he was several times hospitalized because of decompensated heart failure. An implantable cardioverter‐defibrillator (ICD) was implanted 4 months after the posterolateral STEMI. There was no indication for biventricular pacing because the QRS complex was not enlarged. At that time, the renal function was moderately depressed with an eGFR of 40 ml/min per 1.73 m^2^. Because of NYHA Class 3 heart failure with VO2max of only 9 ml/kg/min on maximal ergospirometry, he was considered but not accepted as a candidate for heart transplantation. Four years ago, he was randomized in the sham controlled CHART‐1 clinical trial investigating intramyocardial stem cell injection.^3^ During the study period, there were no hospitalizations for heart failure, and after completion and unblinding, he was informed that he had received active treatment. Two years ago, there was a new series of hospitalizations for heart failure with progressive decline of the renal function. Because of poor diabetic control, he was treated with insulin. Peritoneal dialysis was started 2 years ago but was abandoned because of several episodes of peritonitis. Since 1 year, he is treated with hemodialysis. Despite low blood pressure especially during dialysis, he still received 80 mg of valsartan, 3.75 mg of bisoprolol, and 50 mg of spironolactone. The cardiac function was severely depressed with EF 35% but with chronically high filling pressures, moderate pulmonary hypertension, and moderate functional mitral regurgitation (MR) with effective regurgitant orifice of 23 mm^2^ and a regurgitant volume of 27 ml, considered not sufficient for a mitraclip. After a new episode of worsening heart failure 6 months ago, he underwent a new heart catheterization and coronary angiography showing patent grafts and moderate in stent restenosis of the CX with preserved fraction flow reserve (FFR) and no indication for revascularization. There was severe postcapillary pulmonary hypertension (mean pulmonary artery pressure 40 mmHg) and an elevated wedge pressure of 29 mmHg. The heart rate was 62, he was not considered a good candidate for ivabradine. Because there were no other treatment possibilities, we started a treatment with sacubitril/valsartan. The drug was started on a nondialysis day with a blood pressure of 97/56 mmHg. NT‐proBNP at that time was 11 400 pg/mL. He tolerated the starting dose of 24/26 mg sacubitril/valsartan twice a day, also on dialysis days. After 4 weeks at a follow‐up consultation, the blood pressure was 105/60 mmHg, and the dose was increased to 49/51 mg twice a day, both on nondialysis and dialysis days. He described a symptomatic improvement and started again to leave the house for a small walk. The functional class improved from NYHA 3 to NYHA 2. Functional testing with 6‐min walk test was performed before the start of treatment with a walking distance of 300 m. Because of the development of a diabetic foot ulcer, the test could not be repeated. Filling pressures estimated by echocardiography decreased after the initiation of sacubitril/valsartan (*Table* 1) without changing the intensity of ultrafiltration. There was also a reduction in function mitral insufficiency. Three months after initiation of sacubitril/valsartan, the NT‐proBNP value was reduced to 5,960 pg/ml. Until now, 6 months after the initiation of sacubitril/valsartan, there were no episodes of worsening heart failure.

**Table 1:** Echocardiographic parameters

	3 months before start	Start of treatment	4 months after starting
E wave (cm/s)	97	94	83
A wave (cm/s)	27	38	63
E/A ratio	3,59	2,36	1,3
Deceleration Time (ms)	174	125	171
E/E'	21	22	18
Tricuspid regurgitation gradient (mmHg)	53	57	33
Mitral regurgitation degree	Moderate	Moderate	Mild
ERO (cm^2^)	NA	0,23	0,10
Regurgitant volume (ml)	NA	27	10
Estimated right atrial pressure (mmHg)	15	15	5‐10

Echocardiographic parameters before, at the time of starting, and 4 months after starting sabubitril/valsartan.

## Discussion

There is an unmet need of data on the heart failure treatment with ARNI in patients with heart failure and severe renal insufficiency. The UK HARP‐III trial (United Kingdom Heart and Renal Protection‐III) has demonstrated that, in a wide range of people with proteinuric chronic kidney disease, adding neprilysin inhibition to angiotensin II receptor blockade has no additional effect on kidney function or albuminuria compared with irbesartan.^4^ The tolerability and safety profiles of the two treatments were not different. However, compared with irbesartan, sacubitril/valsartan further reduces both blood pressure and biomarkers of cardiovascular risk (Troponin I and N‐terminal pro‐B‐type natriuretic peptide) and raises the hypothesis that ARNI could reduce cardiovascular morbidity and mortality in a heart failure population with chronic kidney disease. In this study, patients with a GFR from 20 to 60 ml/min per 1.73m^2^ were included.^4^ Yet patients with end stage renal disease and heart failure are not uncommon in daily practice and data on treatment with sacubitril/valsartan in this population are lacking. To our knowledge, there are no reports of hemodialysis patients treated with sacubitril/valsartan.

We describe a patient undergoing hemodialysis with severe end stage systolic heart failure due to ischemic heart disease still symptomatic despite optimal medical treatment with no other therapeutic options. Although the blood pressures were strikingly low, we were able to increase sacubitril/valsartan to the intermediate dose of 49/51 mg twice a day, also on dialysis days. There was a symptomatic improvement with a change from NYHA Class 3 to NYHA Class 2. The chronically elevated filling pressures decreased several months after the initiation of ARNI without changing the intensity of ultrafiltration. Although the interpretation of NT‐proBNP levels is difficult in patients with end stage renal disease, the reduction of NT‐proBNP was impressive and concordant with the clinical improvement.

In conclusion, in this patient with severe heart failure undergoing hemodialysis, treatment with sacubitril/valsartan was feasible, safe, and accompanied by a symptomatic improvement.

## Conflict of interest

Dr Alex Heyse has received a speaker honorarium from the dug company Novartis.

All other authors declare no conflict of interest.
